# Editorial: Indigenous knowledge and chronic disease prevention among the first people of North America

**DOI:** 10.3389/fpubh.2023.1150221

**Published:** 2023-05-24

**Authors:** Melinda S. Smith, Nicolette Teufel-Shone

**Affiliations:** ^1^Interdisciplinary Health, Northern Arizona University, Flagstaff, AZ, United States; ^2^Department of Health Sciences, Northern Arizona University, Flagstaff, AZ, United States

**Keywords:** Indigenous health, Indigenous framework, Indigenous knowledge, community-based participatory research (CBPR), chronic disease treatment and prevention

Indigenous populations of North America face substantial health disparities, which have led to disproportionate rates of chronic diseases, compounded by elevated rates of poverty, lack of educational opportunities, and historical trauma (1, 2). The use of local Indigenous knowledge and culturally-anchored interventions have resulted in sustainable programs that support tribal sovereignty and self-determination (3–5). However, case studies illustrating the role of Indigenous knowledge in changing the course of chronic disease health disparities are limited.

This special topic issue titled *Indigenous Knowledge and Chronic Disease Prevention among the First People of North America*, presents a collection of original research and literature reviews illustrating the role of traditional frameworks and cultural assets to inform the development and implementation of programs and interventions addressing chronic disease prevention and health promotion in Indigenous communities. The ten articles include one mixed studies review, two random clinical trials (RCT) studies, and seven articles that present the development and/or results of culturally-anchored health programs. The mixed studies review included in this issue documents the identification and application of risk and protective factors to inform cancer surveillance and health promotion efforts in Indigenous communities. Some of the interventions described in this issue, engaged traditional knowledge holders in the training of Indigenous and non-Indigenous scholars and researchers. Engaging traditional knowledge holders is key to ensuring interventions are indeed culturally-anchored and support traditional values, beliefs, and practices (6, 7). Ivanich et al. describes an innovative scholar program intended to support the career development of Indigenous scholars. Dreifuss et al. provides an overview of a summer program aimed to expose Indigenous youth to the potential of a public health career through the integration of academic and cultural knowledge to understand and influence health behaviors and perspectives.

In addition to engaging traditional knowledge holders in training researchers and health care professionals, traditional knowledge holders were employed to guide the development of some of the programs and interventions described in this issue. Both Walls et al. and Wilson et al. emphasize that culturally-anchored diabetes programming yields a meaningful approach to disease management that aligns with patients' worldview of self-care. Rink et al. describe the development of a multi-level RCT that builds upon existing community strengths and traditional knowledge and values to reduce sexual and reproductive health disparities in a northwest Indigenous community. Richards et al. presents results from an Indigenous fatherhood health promotion program evaluation guided by a community workgroup that provided cultural expertise and local insight.

Another important emphasis of this issue is the use cultural assets and local resources to support chronic disease prevention. Margariti et al. shared results from their analysis of self-reported data from Indigenous adults enrolled in a sexually transmitted disease (STD) risk reduction program, that recognized the influence of colonizing stressors and used cultural assets as prevention buffers. With a comparable emphasis on cultural strengths, Blue Bird Jernigan et al. share their design and methods for a planned RCT to assess nutrition-related interventions intended to promote food sovereignty and security in an Indigenous community in Oklahoma. Lastly, Fernandez and Beltrán present their analysis of a qualitative health needs assessment that explores the role of participating in cultural dance as a means to strengthen Indigenous identity and understanding of traditional visions of health and wellbeing to ground substance abuse and HIV prevention efforts.

This issue celebrates the use of traditional knowledge and cultural assets in the development and implementation of programs and interventions to address chronic diseases in Indigenous communities. We are confident that this issue will be a valuable resource for public health professionals and researchers aiming to apply innovative strategies to reduce health inequities among Indigenous populations. This collection advocates for the use of Indigenous frameworks and perspectives to improve the health status of Indigenous communities.

## Author contributions

MS and NT-S confirm shared responsibility for drafting, reviewing, and approving the final version of the editorial.
